# Skill mix change between physicians, nurse practitioners, physician assistants, and nurses in nursing homes: A qualitative study

**DOI:** 10.1111/nhs.12601

**Published:** 2019-04-01

**Authors:** Marleen H. Lovink, Anneke (J.A.H.) van Vught, Anke Persoon, Raymond T.C.M. Koopmans, Miranda G.H. Laurant, Lisette Schoonhoven

**Affiliations:** ^1^ Scientific Center for Quality of Healthcare (IQ healthcare), Radboud Institute for Health Sciences, Radboud University Medical Center Nijmegen the Netherlands; ^2^ Faculty of Health and Social Studies HAN University of Applied Sciences Nijmegen The Netherlands; ^3^ Department of Primary and Community Care Radboud Institute for Health Sciences Nijmegen the Netherlands; ^4^ Joachim en Anna Center for Specialized Geriatric Care Nijmegen the Netherlands; ^5^ Julius Center, Nursing Science University Medical Center Utrecht the Netherlands; ^6^ Faculty of Health Sciences University of Southampton Southampton UK

**Keywords:** the Netherlands, nurse practitioner, nursing home, physician assistant, qualitative research, skill mix change

## Abstract

Nursing home physicians face heavy workloads, because of the aging population and rising number of older adults with one or more chronic diseases. Skill mix change, in which professionals perform tasks previously reserved for physicians independently or under supervision, could be an answer to this challenge. The aim of this study was to describe how skill mix change in nursing homes is organized from four monodisciplinary perspectives and the interdisciplinary perspective, what influences it, and what its effects are. The study focused particularly on skill mix change through the substitution of nurse practitioners, physician assistants, or registered nurses for nursing home physicians. Five focus group interviews were conducted in the Netherlands. Variation in tasks and responsibilities was found. Despite this variation, stakeholders reported increased quality of health care, patient centeredness, and support for care teams. A clear vision on skill mix change, acceptance of nurse practitioners, physician assistants, and registered nurses, and a reduction of legal insecurity are needed that might maximize the added value of nurse practitioners, physician assistants, and registered nurses.

## 1 INTRODUCTION

The number of older adults with chronic diseases and multi‐morbidity increases rapidly, resulting in rising pressure on nursing homes (World Health Organization, 2015). Concurrently, few medical students are pursuing a career in health care for the aging population (Golden, Silverman, & Issenberg, 2015). Moreover, quality deficits, such as use of unnecessary restraints, in nursing homes are an issue of international concern (Organisation for Economic Cooperation and Development, 2005). Changing the skill mix by introducing nurse practitioners (NPs), physician assistants (PAs), and baccalaureate‐educated registered nurses (RNs) in nursing homes might diminish physicians' workload and enhance quality of health care (Caprio, 2006; Intrator et al., 2015). These professionals can perform tasks previously reserved for physicians.

In the Netherlands, adults from all ages with complex care needs are entitled to a place in nursing homes. The mean age of nursing home residents was 85 years in 2015/2016 (Verbeek‐Oudijk & Van Campen, 2017). Nursing homes mostly consist of three types of units: units for patients with physical disabilities, dementia special care units, and geriatric rehabilitation units. Multi‐disciplinary teams are employed by the nursing homes, including nursing home physicians (called “elderly care physicians” [ECPs]), nurses, physiotherapists, dieticians, and psychologists (Koopmans, Pellegrom, & Van der Geer, 2017). Elderly care medicine is a unique specialty with a 3 year training program that exists nowhere else in the world (Koopmans et al., 2017).

At present, 1524 ECPs work in Dutch nursing homes, but the vacancy rate is more than 10% (Capaciteitsorgaan, 2016). This shortage is one of the reasons why NPs and PAs were introduced, resulting in approximately 300 NPs and 40 PAs currently employed in nursing homes. In addition, an unknown but relatively low number of RNs work in nursing homes to support ECPs. Actual care is provided by certified nurse assistants and vocationally‐trained RNs (Backhaus, Verbeek, van Rossum, Capezuti, & Hamers, 2018). NPs and PAs are educated at master level, and their title is protected by law; that is, it is reserved for those who have completed a Master of Advanced Nursing Practice or a Master of Physician Assistant, and are registered in their specialty register.

Since 2012, NPs and PAs who are qualified and work in accordance with the legal framework are allowed to independently indicate and perform some of the so‐called “reserved procedures”, which were initially reserved for physicians; for example, giving injections (Freund et al., 2015). RNs are only allowed to perform reserved procedures when showed to be qualified and after instructions from a physician, NP, or PAs (i.e. delegation). While RNs mainly provide nursing care, NPs combine nursing care with medical care, and PAs mainly provide medical care (Ballweg, Sullivan, Brown, & Vetrosky, 2008; Professional Association of Healthcare Professionals – Nurse Practitioners, 2015).

Skill mix change by introducing NPs, PAs, or RNs is achieved through: (i) task delegation from physicians: moving a task to a lower grade provider (physician remains responsible); (ii) physician substitution: expanding the breadth of a job by providing the same services as the physician (new provider is responsible/autonomous); or (iii) physician supplementation: increasing the depth of a job by providing additional services that complement or extend those provided by the physician (Laurant et al., 2018; Sibbald, Shen, & McBride, 2004). In practice, we might see a combination of the three different types of skill mix change in nursing homes (Bakerjian, 2008; Bedin, Droz‐Mendelzweig, & Chappuis, 2013; Rantz et al., 2014).

The shortage of ECPs has generated interest in physician substitution by NPs, PAs, and RNs. It is expected that NPs and PA substitute for ECPs more than RNs, given their level of education, qualifications, and authorizations (Ackermann & Kemle, 1998; Aigner, Drew, & Phipps, 2004). Nevertheless, RNs might sometimes substitute for physicians, as the work of physicians goes beyond reserved procedures; for example, patient assessment (Bedin et al., 2013; Laurant et al., 2018).

A recent systematic review evaluated the effects of physician substitution by NPs, PA, or RNs in nursing homes and primary health care for the aging population. Physician substitution appeared to achieve patient outcomes, such as health status and functional ability, and process of care outcomes, which were at least as good as when care was provided by physicians (Lovink, Persoon, Koopmans, et al., 2017). That review also showed several challenges in introducing NPs, PAs, and RNs, such as physicians' unwillingness to share responsibility for patient care (Lovink, Persoon, Koopmans, et al., 2017). In practice, changes in skill mix are often introduced by government and managers. However, to achieve successful changes in skill mix, it is important to assess the perspectives of the involved professionals (Bryant‐Lukosius & Dicenso, 2004).

### 1.1 Study aim

The aim of this study was to describe how skill mix change in nursing homes is organized from four monodisciplinary perspectives and the interdisciplinary perspective, what influences it, and what its effects are. The study focused particularly on skill mix change through the substitution of ECPs by NPs, PAs, or RNs.

## 2 METHODS

### 2.1 Design

This study was a generic qualitative study using focus group interviews based on a topic list and content analysis (Caelli, Ray, & Mill, 2003; Elo & Kyngas, 2008; Vaismoradi, Turunen, & Bondas, 2013). Focus group interviews provide more information than the sum of individual interviews due to the interaction process (Krueger & Casey, 2009). The COREQ (COnsolidated criteria for REporting Qualitative research) checklist was used for reporting the study (Tong, Sainsbury, & Craig, 2007).

### 2.2 Participants and setting

Four groups were purposefully selected to participate in a monodisciplianry focus group: ECPs (working/have been working with an NP or PA) and NPs, PAs, and RNs working in Dutch nursing homes. In order to identify eligible participants, the professional association/network of each profession contacted its members by email or digital platforms and asked them to fill out a short, self‐developed questionnaire on age, sex, workplace, years of experience, and type of skill mix change. The questionnaire was meant to sample participants per group based on maximum variation (Creswell, 2007). Due to limited response, it was only possible to make a purposeful selection in the group of NPs. The ECPs, PAs, and RNs who filled out a questionnaire were invited for a focus group interview (convenience sampling).

### 2.3 Data collection

For the focus group, the following topics related to skill mix change were discussed: tasks, barriers, facilitators, improvements, and perceived effects (Appendix A). The topics were based on a previous literature review and discussion among the researchers (Lovink, Persoon, Koopmans, et al., 2017). The monodisciplinary focus groups were followed by one interdisciplinary focus group to gain more in‐depth information from an interdisciplinary perspective. This enabled us to discuss the differences of opinions within the various disciplines. The focus group discussions took place at meeting centers, lasted approximately 120 min, and were moderated and observed by two researchers. One of these researchers attended all focus group interviews and a 2 day course on how to conduct focus group interviews prior to the events. Each focus group started with an introduction round, followed by a discussion of the items on the topic list. The observer(s) paid special attention to interactions and non‐verbal communication, made field notes, and asked additional questions, if needed. The moderators and observers all had knowledge of the topic, as they had conducted previous studies about skill mix change. This helped them to stimulate discussions during the focus groups; for example, by asking challenging questions. They were aware of the necessity to keep an open mind while collecting data. They discussed this within the research team and after each focus group. Data were collected from October 2014 to May 2015.

Each participant received an information letter explaining the goal of the study, the interview procedure, and confidentiality of data. Participants of the monodisciplinary focus groups also received additional information about the research questions, definitions of the different forms of skill mix changes, and examples of skill mix change. The participants in the interdisciplinary focus group received a summary of the results of the monodisciplinary focus groups beforehand.

### 2.4 Data analysis

All interviews were audiotaped, transcribed verbatim, and independently coded in Atlas.ti by two researchers. An inductive content analysis was performed, consisting of open coding, creating categories, and abstraction (Elo & Kyngas, 2008; Graneheim & Lundman, 2004; Härkänen, Blignaut, & Vehviläinen‐Julkunen, 2018; Sriyasak, Almqvist, Sridawruang, & Häggström‐Nordin, 2018). Two researchers discussed and compared their codes until consensus was reached. The emerging categories were discussed within the research team.

### 2.5 Rigor

The trustworthiness of the study findings was based on the following criteria: credibility, dependability, confirmation, and transferability (Lincoln & Guba, 1985). Credibility was promoted by the fact that the interview topics were based on a systematic review. In addition, the researchers who conducted the interviews all had knowledge of skill mix change. The interdisciplinary focus group can be viewed as a member check, in which additional viewpoints were discussed. Confirmation was enhanced by extensive discussion of the results within the research team, which includes people with different backgrounds; that is, nurses and ECPs. The results were extensively discussed within the research team. Clear descriptions of the participants and setting, data‐collection procedures, and the process of analysis are presented to promote dependability and facilitate readers' judgements about transferability.

### 2.6 Ethical considerations

The research ethics committee of the region Arnhem Nijmegen concluded that this study did not fall within the scope of the Medical Research Involving Human Subjects Act (registration no. 2014/298) and that the study could be carried out. Written informed consent was obtained from all interviewees at the start of each interview after they received written and verbal information. The confidentiality and privacy of the interviewees and their responses were assured.

## 3 RESULTS

### 3.1 Interviewees' characteristics

In total, 32 providers, who worked for 26 different nursing homes, were interviewed (Table 1). All RNs had additional training in geriatrics or as a practice nurse. The RNs had European Qualification Framework (EQF) 4, 5, or 6. EQF is a European reference framework that aims to make qualifications more readable and understandable across different countries and systems (European Commission, 2016). NPs and PAs had EQF 7, and ECPs had EQF 8.

**Table 1:** Interviewees' characteristics (n = 32)

Focus group participants	Age median (IQR)	Female
***First round***		
NP (*n* = 8)	52 (49–53.5)	*n* = 7
PA (*n* = 5)	52 (48–52)	*n* = 2
ECP (*n* = 7)	53 (37.5–57)	*n* = 5
Practice nurse (*n* = 3) Geriatric nurse (*n* = 1) Nurse in training for specialty in gerontology, geriatrics (*n* = 1)	50 (49–53)	*n* = 5
Second round		
• ECP (n = 2) NP (*n* = 2) PA (*n* = 2)^a^ Practice nurse (*n* = 2)	50.5 (43.3–55.8)	*n* = 4

a One PA also participated in the first round.

ECP = elderly care physician; IQR = interquartile range; NP = nurse practitioner; PA = physician assistant.

### 3.2 Categories

Three main categories emerged from the analysis: variation in skill mix change, factors contributing to variation, and impact despite variation. These categories are interrelated; the different factors contributed to the variation of skill mix change, and despite the variation, skill mix change was perceived to have an impact. The main categories and their subcategories are described (see Table 2 for data excerpts, codes, and category classifications).

**Table 2:** Data excerpts and category classification

Data excerpts	Code	Subcategory	Main category
I work on a dementia special care unit, with 12 beds, and on unit for patients with chronic physical disabilities, also with 12 beds. I'm the medical contact person (PA 2.4) 2 years ago, the board of directors asked all the practice nurses to follow a specialization course at bachelor level, so we all did that. My specialization is care for patients with diabetes mellitus care and nutrition (practice nurse 4.2)	Working at a unit	Unit vs organizational level	Variation in skill mix change
	Having a special area of expertise at the organizational level		
On the medical domain, we re‐invented the wheel, shaped by the elderly care physician who supported me. We have made a delineation of health‐care problems that I am allowed to treat (NPs 1.4)	PA: complex medical tasks	Levels of complexity of tasks	
	NPs: medical tasks varying from “according to protocols” to “complex”		
	RNs: Nursing task and supporting ECPs in medical tasks		
We call or meet ad hoc if they (NPs or PA) want to show or ask me something. When you have a personal connection, interaction is easier. I have been trainer of 4 people, and sometimes this goes well and sometimes it does not go well (ECPs 3.7)	One vs more ECPs	Variation in collaboration with ECPs	
	Structural versus ad‐hoc meetings		
	Working alone versus working in partnership		
	Peer consultation vs supervision		
	Collaborative agreement		
	Trust		
Actually, in our medical team, the idea prevails that the elderly care physician I work in partnership with and who is my supervisor has the final responsibility for everything I do, while in practice, because I work very independent(ly), I'm responsible for everything I do. Actually, I only give it (responsibility) to him if I ask him something or ask to observe something along with me (NPs 1.2)	Legal consequences	Different ideas about responsibilities	
	Final responsibility		
The vision and the spot on the horizon, that was a barrier for me, especially in the beginning, because there was no spot on the horizon (NPs 1.4)	Employment by coincidence	Lack of a vision	Factors contributing to variation
	Vision not a priority		
	Conservative standpoint The Dutch association of ECPs		
Most practice nurses need 3–4 years to eliminate resistance, because actually every nursing assistant or nurse wants to talk to a physician and not to his assistant (practice nurse 4.3) Sometimes I fall on my face terribly. If I, for example, want to consult a cardiologist in our hospital, then I do not even get him on the phone, as the assistants, the people of the outpatient clinic, have the instruction to only forward the call if it is a physician (NPs 1.6) We are educated to think and act like a physician. We are all physicians, it is in our name, and NPs are often employed on a certain specialism (PA 2.5) I think a PA fits better in the hospital, in medical unit care. (NPs 1.3)	Unfamiliarity	Lack of acceptance	
	Struggles in daily practice		
	Issues related to the domain of the other professionals		
I have to say, like you said, it (physician substitution) differs from physician to physician, how open they are to it (physician substitution), I see that too at our place (ECPs 5.1)	Personal characteristics and ideas of ECPs	Personal factors	
	Personal characteristics and ideas of NPs		
	Personal characteristics and ideas of PA		
You have more continuity. Indeed, medical residents, a year, then they are gone (ECPs 3.6) We (NPs) have a broader view. I always give the example: a baker who does not want to sleep anymore at 6 o'clock in the morning, because he is used to wake up at 4 o'clock for 40 years….Then you can give him pills (sleep medication), but I think you have to go where the patient goes, and you have to involve the night shift, give that man something to do….Then you see that our view differs from the view of a physician. A physician would prescribe medication more quickly, so to make him sleep (NPs 1.1) Yes, I perceive that that (shaped at the unit at the bedside) is more accessible and that is not my opinion, that is what the care team says. Yes, because the step to the physician…we have to take in mind that they are the care team; they see the physician as a status symbol (PA 5.5)	Contribution to quality of health care	Added value	Impact despite variation
	Provision of patient‐centered care		
	Support of the care team		
I expect from the future that it (skill mix change) will and has to be introduced more, because if I look at my 35 year career, what my tasks were as physician, I intervened in everything, because there was no one, and I arranged everything. I even knew it when glasses were lost (ECPs 3.1)	Negative side of monitoring role	Changing role of the ECPs	
	Positive side of monitoring role		

ECP = elderly care physician; NPs = nurse practitioner; PA = physician assistant; RNs = registered nurse.

#### 3.2.1 Variation in skill mix change

Skill mix change by introducing NPs, PAs, and RNs was organized in various ways. This category included the following subcategories: unit versus organizational level, levels of complexity of tasks, variation in collaboration with ECPs, and different ideas about responsibilities.

##### Unit versus organizational level

Most NPs, PAs, and RNs took care of older adults at one or more units in the nursing home; for example, units for patients with physical disabilities, dementia special care units, or geriatric rehabilitation units. Several providers also had a special area of expertise at the organizational level; for example, medication safety, physical restraints, wound care, or lung diseases and oxygen use. Most NPs, PAs, and RNs were positioned in the medical team; some RNs in the nursing team.

##### Levels of complexity of tasks

The tasks of NPs and PAs were largely comparable, although PAs performed more complex tasks (related to multi‐morbidity and beyond protocols). Examples of tasks NPs/PAs took over from ECPs were intake of new patients, medical rounds, multi‐disciplinary and family meetings, and prescribing medication. There was little variety in tasks performed by PAs, as they performed quite complex medical tasks. Tasks of NPs varied from only performing tasks according to protocol (delineated) to performing more complex tasks.

The RNs reported to prepare the work for ECPs and support them in medical care. They also provided nursing care. They performed medical tasks, such as preparation before the medical round, assessment of patients in acute situations, monitoring of diabetes mellitus, and prescribing medication (medication was checked by an ECP in advance or afterwards).

##### Variation in collaboration with elderly care physicians

Collaboration with the ECPs varied from collaboration with only one ECPs to a group of ECPs. Some NPs, PAs, and RNs had structural meetings with an ECP; others only had ad hoc meetings.

Most NPs/PAs described the patients they took care of as those they were responsible for; others reported that they worked on the unit in partnership with the ECP. If necessary, NPs/PAs asked an ECP for help, which some called “supervision”, while others called it “peer consultation”. The RNs mainly worked under the supervision of an ECP.

A collaborative agreement between NPs, PA, or RNs and ECPs on prescription of medication, collaboration methods, and responsibilities was deemed to be important to create clarity. Not all nursing homes had such agreements.

Some interviewees reported that trust and personal connection between ECPs and NPs, PAs, or RNs were more important than agreements. All interviewees reported that an NP, PA, or RN should always be able to contact an ECP for consultation by phone or face to face.

##### Different ideas about responsibilities

All providers were concerned about the legal consequences of substituting responsibilities, and added that more information about the legal aspects of doing so was needed. Different opinions were expressed with regard to responsibility of the NPs, PA, or RNs. Some NPs/PA stated that they themselves had final responsibility and were able to perform as head practitioner; others stated that only the ECPs could have final responsibility. Nevertheless, many NPs/PA said they were responsible for their own actions and boundaries. Some ECPs stated they always held final responsibility, others spoke of a shared responsibility, and some were searching for the right division of responsibilities. ECPs thought that PAs could handle greater responsibility than NPs. Finally, most RNs stated that the ECP always had final responsibility.

#### 3.2.2 Factors contributing to variation

Variation in the organization of skill mix change was caused by different factors: lack of a vision, lack of acceptance, and personal factors.

##### Lack of a vision

Reasons to employ an NP, PA, or RN were to substitute for or support ECPs, to improve quality of health care, and to lower expenses. In many cases the decision to employ an NP, PA, or RN was more or less the result of coincidence rooted in external factors, such as grants for training of NPs and PAs, and a shortage of ECPs. A clear vision on roles, tasks, and responsibilities of NPs, PAs, and RNs was lacking, or at least unknown to the interviewees. A vision was considered a prerequisite that would support skill mix change in nursing homes, as it would provide more clarity about its goals and the roles of each provider. Interviewees stated that managers and ECPs did not prioritize the formulation of a vision, due to high pressures on nursing homes. ECPs mentioned that the Dutch association of ECPs had issued guidance on how to organize skill mix change, but the standpoint of the association was conservative, ambiguous, and more reserved than that of the professional association of NPs.

##### Lack of acceptance

Acceptance of the NPs, PAs, or RNs was considered to start with familiarity with their function, tasks, and responsibilities in the nursing home and on a societal level. Many interviewees reported that managers, ECPs, other providers, and patients and their family were ignorant of the function of NPs, PAs, and RNs. Diversity in the types and employment of providers did not contribute to acceptance.

Some PAs were appointed as a medical resident or NPs, which they perceived as an acceptance problem and a reflection of the absence of a vision. NPs, PAs, and RNs often experienced problems if they wanted to contact a hospital physician because some hospital physicians only wanted contact with physicians from the nursing home.

Issues related to the domain of the other provider were present as well. NPs stated that PAs were more medically oriented and they wondered whether PAs without a nursing background were able to work in nursing homes. PAs stated that NPs could only work on one medical specialty and they thought nursing home care was too broad for NPs. In addition, RNs saw a broader role for themselves than did the other interviewees.

##### Personal factors

Providers' personal characteristics and ideas contributed to diversity in the organization of skill mix change. Among ECPs there was diversity in willingness to share responsibility, level of experience, and personality. Among NPs there was diversity in level of preceding experience, level of autonomy, and personality. An extra variety among PAs appeared to be their professional educational background (e.g. physiotherapist, nurse). These differences made it necessary to seek harmonization within each individual collaboration between ECPs and NPs/PAs. This collaboration had to grow over time. Diversity among RNs seemed not to be an issue.

#### 3.2.3 Impact despite variation

The introduction of NPs, PAs, and RNs was perceived to have an impact on “added value” and “the role of the ECPs”, although skill mix change was organized differently.

##### Added value

The contributions of the NPs, PAs, and RNs to the quality of health care, provision of patient‐centered care, and support of the care team were perceived to be an added value.

NPs, PAs, and RNs were reported to contribute to quality of health care by improving continuity of care, registration of the ECPs in the medical record became more structured, and quality improvement projects took place.

NPs and RNs perceived that they distinguished themselves from ECPs on the nursing domain, which contributed to improved patient‐centered care, because they knew their patients very well, involved family, leveled with patients and family during conversations, were accessible to patients and family, had an overall view of the patient, worked by means of a process, and took/had time for patients and family. PAs also reported that they improved patient‐centered care because of their interest in patients, accessibility and time for patients and family.

Participants perceived that NPs, PAs, and RNs supported the care team because they were accessible, and coached, educated, and trained the team, reminded them of their own responsibilities, positioned themselves next to and not above them, took them seriously, took/had time for them, understood the daily practice, and had an exemplary role.

##### Changing role of the elderly care physicians

The role of ECPs changed with the introduction of NPs, PAs and RNs from a more practical role to a role further away from patients to a coordinator. Some ECPs did not like this role; they stated that they missed patient contact and risked losing their skills. Some ECPs said that they had more time for other tasks, such as complex care, workgroups, and tasks in primary health care. Several ECPs stated the negative effect of having more night and weekend shifts (because most NPs, PAs, and RNs did not do those shifts).

## 4 DISCUSSION

The aim of this study was to describe how skill mix change in nursing homes is organized from four monodisciplinary perspectives and the interdisciplinary perspective, what influences it, and what its effects are. This study focused particularly on skill mix change through the substitution of ECPs by NPs, PAs, or RNs. Great variation in skill mix change was found. Despite this variation, stakeholders reported increased quality of health care and a new role for ECPs. A clear vision on skill mix change in nursing homes was missing. Skill mix change was further influenced by lack of acceptance of NPs, PAs and RNs by colleagues and patients, and by providers' personal ideas.

The result confirmed the findings of other studies: that skill mix change in nursing homes shows great variety and is still evolving (Intrator et al., 2015; Lovink, Persoon, Koopmans, et al., 2017; Martin‐Misener et al., 2015). All NPs, PAs, and RNs worked at the unit level. PAs took over a broad range of (complex) tasks from ECPs. Among the NPs, there was a range from only performing tasks according to protocols to performing more complex tasks. The RNs reported that they prepared work for ECPs and supported them in medical care. Some NPs, PAs, and RNs also worked at the organizational level with a special area of expertise. The interviewees described great variation in how NPs, PAs, or RNs and ECPs collaborated and in (ideas on) division of responsibilities. Discussions among interviewees were hindered by their confusion about the meaning of substitution, delegation, and responsibility, and the legal consequences of substituting responsibilities. Therefore, it was difficult to specifically describe the ECPs substitution, as this study aimed to do.

Despite the variation in skill mix change, NPs, PAs, and RNs were considered an added value. A recent systematic review already showed that physician substitution in health care for the aging population appeared to achieve at least as good patient and process outcomes as care provided by physicians (Lovink, Persoon, Koopmans, et al., 2017). The current study revealed that NPs, PA, and RNs add to good patient care by their contributions to quality of health care, provision of patient‐centered care, and strengthening of the care team. In addition, the role of the ECP changed after the introduction of an NP, PA, or RN into a more coordinating and supporting role.

In line with findings of other studies, this study showed that introducing NPs, PAs, or RNs into nursing homes is influenced by factors at the social, organizational, and professional levels (Lovink, Persoon, Koopmans, et al., 2017; Lovink et al., 2018; Niezen & Mathijssen, 2014). To overcome the problems related to role clarity and acceptance and to diminish the influence of personal factors it is important to enter into dialogue at these different levels (Bryant‐Lukosius & Dicenso, 2004). At the professional societal level, the current study showed the need for a joint agreement on skill mix change between the national association of ECPs and the professional associations of NPs, PAs, and RNs (Lovink, Persoon, Koopmans, et al., 2017; Niezen & Mathijssen, 2014). At the organizational level, the current study showed that a vision on skill mix change was lacking or at least unknown to the interviewees. Bryant‐Lukosius and Dicenso (2004) developed a framework for organizations to help them develop a vision on the roles, tasks, and responsibilities of advanced practice nurses in relation to patients' needs, called “the spot on the horizon”. Organizations should answer questions about which profession will be employed, in which manner, and with what purpose. These questions should be answered together with important stakeholders, such as providers themselves, to gain support for skill mix change.

Finally, the current study showed that the tasks and responsibilities of each NP, PA, or RN should be discussed and recorded in dialogue with the collaborating ECP(s). Among the interviewees in this study, there was ignorance and insecurity regarding transferring responsibilities. For example, they talked about final responsibility, while all providers, regardless of level, are responsible for their own actions. Literature suggests that a collaborative agreement is important to create clarity (American Medical Directors Association Ad Hoc Work Group, 2011; Caprio, 2006; O'Brien, Martin, Heyworth, & Meyer, 2009). However, the details about what such an agreement should include are a point of contention. Some believe it should focus on the process (American Medical Directors Association Ad Hoc Work Group, 2011), while others state that it should focus on specific behaviors by specific providers (O'Brien et al., 2009).

Compared to other international studies, our study is unique in combining the perspective of all providers involved in skill mix change, by first conducting monodisciplinary focus group interviews and then bringing professionals together in an interdisciplinary focus group interview. This led to an in‐depth providers' perspective on the way skill mix change in nursing homes is organized. Some limitations should be considered while interpreting the results of this study. First, self‐reporting of activities might lead to social desirability bias and might influence the credibility of the results (Holtgraves, 2004). Interviewees might have described their tasks and responsibilities in ways that did not reflect their true practice. In addition, it was our goal to gain insight into the perspective of the providers, but it would be interesting to combine the perspectives of providers, managers, and patients to enhance credibility. To gain insight into these perspectives and the role of NPs, PAs, and RNs in real practice, a case study using different data‐collection methods, including observations, could be carried out (Lovink et al., 2017). Second, the organization of Dutch nursing homes differs from other countries, which might hamper transferability of the results. In the Netherlands, ECPs are employed by the nursing home and are able to support NPs, PAs, and RNs. It is unclear how other models would influence the role of NPs, PAs, and RNs. For example, geriatricians who provide care to nursing home residents in addition to their primary job in a hospital might be less present, and therefore, unable to fully support NPs, PAs, and RNs in nursing homes (Katz, Karuza, Intrator, & Mor, 2009).

## 5 CONCLUSIONS

Skill mix change by introducing NPs, PAs, and RNs was organized in various ways. Despite this variation, interviewees considered NPs, PAs, and RNs to be an added value to health‐care delivery. The introduction of these professionals changed the role of ECPs, mainly into a more coordinating role, with a focus on complex medical care issues. A clear and shared vision for roles, tasks, and responsibilities of NPs, PAs, and RNs was needed. A shared vision can contribute to greater acceptance of these providers and diminish the influence of personal factors of individual providers on how skill mix change is organized. It is important that all providers involved in skill mix change participate in discussions about vision, and making clear the definitions of substitution, delegation and the legal consequences of transferring responsibilities. Finally, a well‐crafted vision might maximize the added value of NPs, PAs, and RNs, and optimize the role of ECPs.

## AUTHOR CONTRIBUTIONS

Study design: M.L.A, A.V.V., A.P., R.K., M.L.A., and L.S.

Data collection: M.L.A., A.V.V., and A.P.

Data analysis: M.L.A. and A.V.V.

Manuscript writing and revisions for important intellectual content: M.L.A., A.V.V., A.P., R.K., M.L.A., and L.S.

## Interview guide monodisciplinary focus groups interviews

1. What are your tasks in the nursing home?

- How does your position/occupation relate to the position/occupation of other professionals?
- What is your role in relation to other professionals?
- Who performs which tasks?
- Would you describe your tasks as substitution, delegation or supplementation?

2 What is the effect of skill mix change?
3 What are barriers and facilitators to skill mix change?

- What are chances, challenges, threats, conditions, and boundaries for skill mix change?

4 How should skill mix change be organized in the future?
5 What is your role in the future?

- Is it possible that another professional performs your tasks?
- Is it possible that you take over tasks from another professional?
- What will your position/occupation look like in 5 or 10 years?
- Who should perform which tasks?

## Interview guide interdisciplinary focus group interview

Interviewees received a summary of the findings of the first round of (focus group) interviews beforehand.

1. Do you recognize the results of the first round of (focus group) interviews? Are the results complete?
2. What is the optimal model of skill mix change (for the patient) in what circumstances?

- Why should skill mix change be organized in this way?
- Which professionals work together?
- What is the goal of skill mix change?

3 Why is the optimal model of skill mix change not yet a reality?

Topics to discuss:

- Tasks
- Responsibilities
- Effects of skill mix change
- Barriers and facilitators to skill mix change
